# Occurrence of a New Variant of *Salmonella* Infantis Lacking Somatic Antigen

**DOI:** 10.3390/microorganisms11092274

**Published:** 2023-09-10

**Authors:** Alessandra Alessiani, Gianfranco La Bella, Adelia Donatiello, Gilda Occhiochiuso, Simona Faleo, Antonella Didonna, Luigi D’Attoli, Patrizia Selicato, Carmine Pedarra, Giovanna La Salandra, Maria Emanuela Mancini, Pietro Di Taranto, Elisa Goffredo

**Affiliations:** 1 Istituto Zooprofilattico Sperimentare della Puglia e della Basilicata, Via Manfredonia 20, 71121 Foggia, Italy; 2 Istituto Zooprofilattico Sperimentale dell’Abruzzo e del Molise, Via Campo Boario 1, 64100 Teramo, Italy

**Keywords:** *Salmonella* Infantis, *Salmonella* somatic antigen, serotyping, multidrug resistance

## Abstract

*Salmonella* Infantis is one of the most frequent serovars reported in broilers and is also regularly identified in human salmonellosis cases, representing a relevant public health problem. In the laboratories of the Istituto Zooprofilattico Sperimentale della Puglia e della Basilicata (IZSPB), six *Salmonella* Infantis strains with antigenic formula -:r:1,5 have been isolated from the litter and carcass of broilers between 2018 and 2022. The strains were investigated to evaluate their phenotype, antibiotic resistance and genomic profiles. Genomic analysis confirmed that the isolates belonged to the Infantis serotype and to the sequence type ST32. Moreover, all strains showed a multidrug-resistant (MDR) profile and were characterised by the presence of the IncFIB plasmid incompatibility group. Three strains had the *bla*_CTX-M-1_ gene, and one of them carried IncX1. The presence of this new variant of *S*. Infantis is particularly relevant because it could expand the landscape of the *S*. Infantis population. The absence of the somatic antigen could pose a problem in both isolation and serotyping and a consequent public health concern due to the spread of *Salmonella* infection.

## 1. Introduction

*Salmonella* spp. is a major cause of food-borne outbreaks in the EU and the second leading cause of gastrointestinal infection reported in humans [1].

Symptoms of salmonellosis include gastroenteritis accompanied by nausea, vomiting, abdominal cramps, bloody diarrhoea, headache, feverish conditions, and myalgia. Human salmonellosis is usually characterised by self-limiting disease and does not require antimicrobial treatment. However, the infection can be more serious with salmonellosis-related deaths in the very young, the elderly, and immunocompromised people [2].

*Salmonella* can be divided into two species: *Salmonella enterica* and *Salmonella bongori*. Further, *S. enterica* has six subspecies: *enterica* (I), *salamae* (II), *arizonae* (IIIa), *diarizonae* (IIIb), *houtenae* (IV), and *indica* (VI) [2].

*S*. Infantis (6,7:r:1,5) is a *Salmonella enterica* serovar and, with *S*. Enteritidis (1,9,12:g,m:-), *S*. Typhimurium (1,4,[5],12:i:1,2), monophasic *S*. Typhimurium (1,4,[5],12:i:-) and *S*. Derby (1,4,[5],12:f,g:[1,2]), belongs to the five most frequently reported *Salmonella* serovars in human salmonellosis cases acquired in the EU in 2020 [3]. It accounted for 31.5% of the human infections from food-animal sources that occurred in the EU in 2020. It was strictly related to broiler sources (94%), including broiler flocks and broiler meat [1]. More than 50% of the *S*. Infantis isolated in 2019 from broilers was reported in Italy [4].

*Salmonella* serotyping is the traditional method used for the classification, characterisation and surveillance of *Salmonella* worldwide. *Salmonella* serotyping is important in both human and veterinary diagnostics.

Serotyping is based on the agglutination of somatic antigen (O antigen) and flagellar antigens (H antigens) with specific O and H antisera. Combinations of the O and H antigens can divide *Salmonella* enterica into more than 60 serogroups and 2600 serotypes.

In recent years, whole genome sequencing (WGS) has been increasingly used to characterise bacterial isolates for research, outbreak detection, and surveillance. WGS has enhanced studies about genomic diversity among *Salmonella*, allowing us to identify and subtype strains and to assess their relatedness. In addition, WGS can be useful to streamline the laboratory process and reduce processing and turnaround times [5].

Commonly, molecular characterisation associated with serotyping can provide more detailed information on the strains, as recommended by EFSA [6].

Over recent years, antimicrobial resistance has emerged in *S*. Infantis strains from different animal sources and humans in various European countries, leading to complications in disease [7,8]. The spread of multidrug-resistant (MDR) variants is likely due to the widespread and long-term use of common antimicrobials in poultry and animal farms for therapeutics, prophylaxis, and growth promotion [9]. MDR *S*. Infantis strains isolated in broilers have been registered in different countries, such as Hungary, Germany, Italy, the Netherlands, Russia, and the United States [3]. Recent studies highlight that 70% of *S*. Infantis isolated from broiler meats in EU member states in 2016 were MDR [10]. The study focuses on the analysis of *S*. Infantis strains, showing a particular phenotype (lacking somatic antigen), isolated during a four-year period between 2018 and 2022 in the Apulia and Basilicata regions (South Italy). The isolates have been investigated in order to characterise their phenotypic and genotypic profiles and to estimate their resistance to different antimicrobials.

## 2. Materials and Methods

### 2.1. Bacterial Strains

Isolation was performed according to ISO 6579-1:2017 [11] and ISO/TS 6579-2:2012 [12] in the laboratories of the IZSPB as part of official control activities between 2018 and 2022. In detail, a volume of the non-selective enrichment broth Buffered Peptone Water (BPW) (Microbiol, Cagliari, Italy) was added to each sample (1:10 *w*/*v* or *v*/*v*) and then incubated at 34–38 °C for 18 h. Then, 0.1 mL and 1 mL of the culture were transferred in 10 mL of Rappaport Vassiliadis Soy (RVS) broth (Microbiol, Cagliari, Italy) and in 10 mL of Muller–Kauffmann Tetrathionate-Novobiocin (MKTTn) broth (Microbiol, Cagliari, Italy), respectively. The RSV broth was incubated at 41.5 °C for 24 h whilst the MKTTn broth at 34–38 °C for 24 h. After incubation, the obtained culture from each tube was seeded with a sterile inoculation loop (size 10 µL) on the selective agar media Xylose Lysine Desoxycholate (XLD) (Microbiol, Cagliari, Italy) and *Salmonella* Detection Agar (SDA) (Microbiol, Cagliari, Italy) to isolate *Salmonella*. The suspect colonies of *Salmonella* were sub-cultured for identity confirmation. In particular, the isolates were identified as *Salmonella* based on the urea test, the lysine test, and triple-sugar iron (TSI) fermentation. The presence of *Salmonella* was confirmed with biochemical tests API20E^®^ (Biomerieux, Lyon, France) and serological tests. Serotyping was performed by slide agglutination according to ISO/TR 6579-3:2014 [13] using SSI antisera (SSI Diagnostica, Hillerød, Denmark). For each sample, only one *Salmonella* strain was stored at −20 °C in Microbank™ (Pro-Lab Diagnostics, Richmond Hill, ON, Canada).

#### *Salmonella* Strains -:r:1,5

The strains were sent to the National Reference Center for Salmonellosis of the Istituto Zooprofilattico Sperimentale delle Venezie (IZSVE) to confirm the absence of the somatic antigen. The smoothness of the colonies was further evaluated using crystal violet method [14].

### 2.2. Antimicrobial Susceptibility Testing

The strains of *Salmonella* without somatic antigen were characterised phenotypically via broth microdilution using Sensititre^®^ EUVSEC3^®^ plates (Termofisher Scientific, Paisley, UK). The antibiotics on the EUVSEC3^®^ plate were ampicillin (1–32 µg/mL) (AMP), azithromycin (2–64 µg/mL) (AZY), amikacin (4–128 µg/mL) (AMK), gentamicin (0.5–16 µg/mL), tigecycline (0.25–8 µg/mL) (TGC), ceftazidime (0.25–8 µg/mL) (CAZ), cefotaxime (0.25–4 µg/mL) (CTX), colistin (1–16 µg/mL) (CST), nalidixic acid (4–64 µg/mL) (NAL), tetracycline (2–32 µg/mL) (TET), trimethoprim (0.25–16 µg/mL) (TMP), sulfamethoxazole (8–512 µg/mL) (SUL), chloramphenicol (8–64 µg/mL) (CHL), meropenem (0.03–16 µg/mL) (MEM) and ciprofloxacin (0.015–8 µg/mL) (CIP). The quality control of the batch was performed with *Escherichia coli* ATCC^®^ 25922. The antimicrobial test was carried out according to the manufacturer’s instructions. The definition of sensitivity or resistance was based on the Epidemiological Cut Off (ECOFF) [15,16]. The strains were referred to as multi-drug-resistant (MDR) when they simultaneously showed resistance to at least three different classes of antibiotics [17].

### 2.3. In Silico Sequencing and Analysis

The DNA of the strains without somatic antigen was extracted using the DNeasy^®^ Blood & Tissue kit (Qiagen GmbH, Hilden, Germany) following the kit instructions. Genomic sequencing was performed using the MinIONTM platform (Oxford Nanopore Technologies, ONT, plc., Oxford Science Park, OX4 4DQ, UK). The libraries were prepared using the Rapid Barcoding Kit (SQK-RBK004) according to the manufacturer’s protocol. Size selection and clean-up steps were performed using AMPure XP (Beckman Coulter, Brea, CA, USA). The samples were sequenced using the MinKNOW software (ONT, ver. 21.11.6), and the generated Fast5 sequences were analysed using Guppy software (ONT, ver. 4.4.1) with default parameters in order to obtain fastq files. The sequences were analysed and assembled using the Flye software (ver. 2.9) [18]. The genomes were analysed in silico for the identification of serotype, sequence type, genes responsible for antibiotic resistance and groups of plasmid incompatibility using the online tools provided by the Center for Genomic Epidemiology, Technical University of Denmark (DTU): SeqSero 1.2 [19], MLST 2.0 [20], ResFinder 4.1 [21] and PlasmidFinder 2.1 [22], respectively “https://www.genomicepidemiology.org/ (accessed on 1 February 2023)”. This Whole Genome Shotgun project was deposited at the NCBI GenBank under BioProject number PRJNA875381.

## 3. Results

### 3.1. Bacterial Strains

Five hundred and two samples were analysed between 2018 and 2022. One hundred and sixty-eight *Salmonella* Infantis strains were isolated (33.5%). Among these strains, six (3.6%) were *Salmonella* without somatic antigens (-:r:1,5). The samples from which the strains, without somatic antigen, were isolated were collected from five different farms (named A, B, C, D and E) and one market (named M) in the prefectures of Foggia and Lecce in the Apulia region, as shown on Table 1.

The six strains showed typical aspect on the growth media, producing H2S and smooth colonies. They did not show agglutination for any antiserum, for saline and for the antiserum for capsular antigen Vi. The strains were serotyped by the IZSPB laboratory and confirmed by the laboratory of IZSVE as -:r:1,5.

### 3.2. Antimicrobial Susceptibility Testing

The six strains tested for antimicrobial resistance were resistant to amikacin, trimethoprim, tetracycline, sulfamethoxazole and nalidixic acid, falling within the definition of MDR. All were sensitive to meropenem, tigecycline, chloramphenicol, gentamicin and colistin. Two strains were resistant to ceftazidime (33.3%) and three to cefotaxime and ampicillin (50%). Five were resistant to ciprofloxacin (83.3%), as shown in Table 1.

### 3.3. In Silico Sequencing and Analysis

Genome analysis confirmed the belonging of all isolates to the *S*. Infantis serotype antigenic profile -:r:1,5, and the MLST analysis showed that all strains belonged to Sequence Type ST32. The bioinformatic analysis confirmed the presence of three ESBL-producing strains (ID 79, 147, 152), all carrying the *bla*_CTX-M-1_ gene. The presence of genetic determinants responsible for antibiotic resistance was corresponding to the observed phenotypic profile. The *tet*(A), *sul1*, *dfrA1* and/or *dfrA14*, *qacE* genes, conferring resistance to tetracycline, sulfamethoxazole, trimethoprim and quaternary ammonium resistance, respectively, were detected in all isolates. The *aph(3′)-Ia* gene was detected on the plasmid IncFIB in four isolates that were aminoglycoside resistant. The chromosomal gene *aac(6′)-Ia*, conferring aminoglycoside resistance, was detected in all isolates. All strains showed plasmid incompatibility group IncFIB, with the *bla*_CTX-M-1_ gene located on it in ESBL-producing strains. Moreover, strain ID 147 showed a second plasmid belonging to the IncX1 group. The presence of chromosomal point mutations on *gyrA* and *parC* genes confirmed the phenotypic quinolone resistance.

The results of genomic analysis are shown in Table 2.

## 4. Discussion

Over the last few years, *S*. Infantis has dramatically increased in chicken meat, reaching 45% of strains isolated from broiler sources. A slight increase, from 2.3% in 2018 to 2.5% in 2020, was reported among human cases in the EU [3,23,24].

A recent study carried out by IZSVE reported that *S*. Infantis is frequently detected in the litter and in the environmental dust along the broiler chicken supply chain. Moreover, it is extremely persistent, causing relapses in the farms where it is isolated [24]. The relevance of *S*. Infantis is mainly due to the multidrug-resistant strains, which have quickly increased from 20% to 80%. This can be considered a matter of concern for One Health, also due to the presence of pESI-like megaplasmid [3,25,26,27].

From 2018 to 2022, during the IZSPB activity, 30 bacterial strains (5 for each sample) with antigenic formula -:r:1,5 were isolated and serotyped. Somatic antigens are part of the outer membrane (LPS) of *Salmonella* and are both discriminative and generic. Over time, the ability to identify specific antigens has enabled to group *Salmonella* into over 50 serogroups, described in the Kauffmann–White scheme. The differences among somatic antigens seem to be related to the presence of particular prophages that induce their expression [28]. The difficulties usually encountered in *Salmonella* typing can be attributed to the presence of capsular antigen or to a stressful isolation environment. Moreover, it can also be due to the roughness of the colony. Usually, the expression of the rough morphotype called RDAR (red, dry and rough) is due to exposure to challenging environmental factors, such as the presence of disinfectants and suboptimal temperature. Furthermore, the presence and the expression of essential genes, such as *csgD* and *adrA* genes, can be involved in exhibiting the RDAR morphotype. This kind of morphotype is common in *Salmonella* strains isolated in “poultry houses” because it provides resistance to NaOCl and increases the ability to withstand stressful environmental conditions [29,30]. Although the genome sequences of the six investigated strains showed the presence of both genes, *csgD* and *adrA*, the colonies did not have a rough shape and dry texture. The smoothness was confirmed by the staining indicated by White et al. [14]. The detection of *Salmonella* lacking somatic antigen, with a typical aspect of colonies on growth media, was a relevant finding. This serotype was first detected in the litter and subsequently on a carcass, expanding the population variability of *Salmonella* Infantis.

The affiliation to “Infantis serotype” was confirmed with SeqSero. Genome analysis revealed that the six *S*. Infantis strains belong to sequence type ST32, which is very common in Europe and Italy [3,31]. A relevant aspect of these strains is the antibiotic resistance. The simultaneous detection of resistance genes and plasmid incompatibility group IncFIB supports the opinion that these strains were a variant of *S*. Infantis. The presence of the IncX1 plasmid indicates the persistence over time of the studied strains. In fact, this kind of incompatibility plasmid has been repeatedly detected in bacteria of the same ecological niche, such as *Klebsiella*, *Shigella* and *E. coli* [32].

Finally, the presence of the IncFIB plasmid associated with the *bla*_CTX-M-1_ gene and ST32 may suggest that the strains could belong to Cluster I, as indicated in a recent work by Di Marcantonio et al. They characterised *S*. Infantis strains isolated between 2017 and 2020 in the Abruzzo and Molise regions and compared them with Italian *S*. Infantis sequences deposited in national databases, finding that most of the strains belonged to Cluster I [3].

## 5. Conclusions

The results of this present work show that the examined strains are new variant of *S*. Infantis.

The reports of somatic antigen absence are rare. They are demonstrated in relation to roughness or structural damage [33]. In our case, no structural damage can be suspected, and the population is assumed to be stable over time. When a deeper investigation (phenotypic and/or molecular) is not available, it could be difficult to identify the new *S*. Infatis variant, increasing the risk of human infection. Further research will be needed to study the molecular mechanisms that lead to the loss of somatic antigen expression and its effect on the persistence in the environment, such as resisting disinfectants, developing antibiotic resistance, and potentially acquiring virulence.

## Figures and Tables

**Table 1:** Phenotypic resistance profiles and source of investigated strains.

Strain ID	Year	Source	Phenotypic Resistance Profile
74	2022	Carcass/Broiler/Market	AMK-TET-TMP-SUL-NAL-CIP
79	2019	Litter/Broiler/Farm D	AMK-TET-TMP-SUL-NAL-AMP-CIP-CAZ-CTX
86	2018	Litter/Broiler/Farm A	AMK-TET-TMP-SUL-NAL-CHL
147	2018	Litter/Broiler/Farm C	AMK-TET-TMP-SUL-NAL-AMP-CIP-CAZ-CTX
152	2019	Litter/Broiler/Farm E	AMK-TET-TMP-SUL-NAL-AMP-CIP-CTX
203	2018	Litter/Broiler/Farm B	AMK-TET-TMP-SUL-NAL-CIP

**Table 2:** Genetic features of *Salmonella* Infantis strains.

Strain ID	ST	Acquired Resistance Genes	Chromosomal Point Mutation	Plasmid
74	32	*aph(3′)-Ia*, *tet(A)*, *sul1*, *dfrA1*, *dfrA14 qacE*	*gyrA (D87G)*, *parC(T57S*, *E84K)*	IncFIB
79	32	*aph(3′)-Ia*, *tet(A)*, *dfrA1*, *dfrA14*, *bla*_CTX-M-1_, *sul1*, *qacE*	*gyrA(D87G)*, *parC(T57S*, *E84K)*	IncFIB
86	32	*aph(3′)-Ia*, *tet(A)*, *sul1*, *dfrA1*, *dfrA14*, *qacE*	*gyrA(D87G)*, *parC(T57S)*	IncFIB
147	32	*aph(3′)-Ia*, *tet(A)*, *dfrA1*, *dfrA14*, *bla*_CTX-M-1_, *sul1*, *qacE*	*gyrA(D87G)*, *parC(T57S*, *E84K)*	IncFIB, IncX1
152	32	*tet(A)*, *dfrA1*, *dfrA14*, *bla*_CTX-M-1_, *sul1*, *qacE*	*gyrA(D87G)*, *parC(T57S*, *E84K)*	IncFIB
203	32	*tet(A)*, *sul1*, *dfrA1*, *dfrA14*, *qacE*	*gyrA(D87G)*, *parC(T57S*, *E84K)*	IncFIB

## Data Availability

The data presented in this study are openly available and are deposited at the NCBI GenBank under BioProject number PRJNA875381.
